# Case report: Acute necrotizing encephalopathy: a report of a favorable outcome and systematic meta-analysis of outcomes with different immunosuppressive therapies

**DOI:** 10.3389/fneur.2023.1239746

**Published:** 2023-09-01

**Authors:** Stefanie Zaner Fischell, Jonathan Fischell, Tamara Kliot, Jamie Tumulty, Stephen J. Thompson, Madiha Q. Raees

**Affiliations:** ^1^Department of Pediatrics, University of Maryland Medical Center, Baltimore, MD, United States; ^2^Division of Pediatric Neurology, Department of Pediatrics and Neurology, University of Maryland School of Medicine, Baltimore, MD, United States; ^3^Division of Critical Care, Department of Pediatrics, University of Maryland School of Medicine, Baltimore, MD, United States

**Keywords:** acute necrotizing encephalopathy, immunosuppressive therapy, outcomes, autoimmune diseases of the nervous system, pediatric neurology, meta-analysis

## Abstract

Acute Necrotizing Encephalopathy (ANE) is a condition characterized by symmetric, bilateral lesions affecting the thalamus and potentially other areas of the brain following an acute febrile illness. It manifests clinically as abrupt development of encephalopathy, or alteration in mental status that often includes development of seizures and progression to coma. Treatment strategies combine immunosuppressive therapies and supportive care with varying levels of recovery, however there are no universally accepted, data-driven, treatment algorithms for ANE. We first report a case of a previously healthy 10-year-old female with acute onset diplopia, visual hallucinations, lethargy, and seizures in the setting of subacute non-specific viral symptoms and found to have bilateral thalamic and brainstem lesions on MRI consistent with ANE. She was treated with a combination of immunomodulatory therapies and ultimately had a good outcome. Next, we present a meta-analysis of 10 articles with a total of 158 patients meeting clinical and radiographic criteria for ANE. Each article reported immunosuppressive treatments received, and associated morbidity or mortality outcome for each individual patient. Through our analysis, we confirm the effectiveness of high-dose, intravenous, methylprednisolone (HD-IV-MP) therapy implemented early in the disease course (initiation within 24 h of neurologic symptom onset). There was no significant difference between patients treated with and without intravenous immunoglobulin (IVIG). There was no benefit of combining IVIG with early HD-IV-MP. There is weak evidence suggesting a benefit of IL-6 inhibitor tocilizumab, especially when used in combination with early HD-IV-MP, though this analysis was limited by sample size. Finally, plasma exchange (PLEX) improved survival. We hope this meta-analysis will be useful for clinicians making treatment decisions for patients with this potentially devastating condition.

## 1. Introduction

Acute necrotizing encephalopathy (ANE) is an acute neuroinflammatory condition characterized by selective, symmetric brain lesions affecting the bilateral thalami with or without other brain involvement (1–4). First described in 1995 by Dr. Mizuguchi in Japanese and Taiwanese children presenting with new onset focal neurological deficits, seizures, and altered mental status following a viral illness. Common viruses include, but are not limited to influenza A, influenza B, parainfluenza, and various members of the herpes and enterovirus families (3, 5–7). While the exact pathogenesis is not fully understood, evidence suggests that ANE results from an exaggerated systemic inflammatory response characterized by extensive release of pro-inflammatory cytokines and natural killer cell activation (6). Brain involvement is thought to result from blood brain barrier disruption from both cytotoxic and vasogenic edema causing confusion, altered level of awareness, and potentially focal neurologic deficits or seizures (6, 8). While most cases are sporadic, familial cases of ANE have been associated with a causative gene mutation (*RANBP2*) (9). Brain magnetic resonance imaging (MRI) demonstrates symmetric T2 hyperintense lesions in the bilateral thalami along with the brainstem, periventricular white matter, and cerebellum; presence of brainstem lesions portend a worse prognosis (1, 6, 8, 10).

There are no standardized treatment guidelines for ANE; however, generally utilized treatment strategies combine immunosuppressive interventions and supportive/symptomatic management. The most frequently reported immunosuppressive therapy is high dose intravenous (IV) steroids [methylprednisolone, (MP) at least 30/mg/kg/day for at least 3 days] (8, 10–14). Additionally, case reports and case series report the use of intravenous immune globulin (IVIG) (10, 15, 16), plasma exchange (PLEX) (10, 16–19), and biologics such as tocilizumab (an IL-6 inhibitor) (8, 11, 19, 20). Supportive care and symptomatic management in an intensive care unit (ICU) is necessary in most cases for depressed mental status, seizure control, blood pressure support, multisystem organ failure management which often requires intubation (3, 8, 16, 19).

While rare, ANE is an acute disease with devastating sequelae that affects otherwise healthy children. Morbidity and mortality is estimated to be between 60–90% and 20–40%, respectively (1, 2, 11, 16). Since its discovery, many groups have contributed to our growing understanding of both diagnosis and treatment. In 2009, immunosuppressive therapies became standard after a landmark paper reported their benefit in 2009 (14). However, not all patients benefit from early steroids alone, leading to the exploration of adjunctive therapies (13–15). Case reports of patients treated with IVIG, PLEX, and tocilizumab have shown promise with good outcomes. It is important to acknowledge that these therapies need to be studied in larger patient populations to acquire statistically meaningful data to support their use. While considerable advances have been made to improve the management of ANE, morbidity and mortality remains high (10, 16, 21). A scoring system to predict ANE severity (ANE-SS) was created in 2015 based on available literature (17).

In this report, we describe the case of a 10-year-old who with severe ANE (based on ANE-SS), including brainstem involvement, who made a near-full recovery. While seeking guidelines to treat this patient, we recognized the paucity of evidence-based recommendations for management of ANE. While it has been demonstrated that steroid initiation within 24 h of neurologic worsening is beneficial, few have analyzed outcomes with other treatment modalities such as IVIG, tocilizumab, or combination therapies. Especially because the morbidity of ANE is so high, it is imperative to offer patients optimal, data driven immunosuppressive treatment regimens. In this manuscript, we provide compiled outcome data from 10 papers which reported immunosuppressive therapies and subsequent outcomes in patients with ANE. We hypothesize that additional treatment modalities beyond early steroids (IVIG, PLEX or tocilizumab) lead to improved morbidity outcomes, especially in patients with more severe disease.

## 2. Methods

### 2.1. Case report

The case report is a written report of a patient’s course in the Pediatric ICU at the University of Maryland Medical Center in 2020.

### 2.2. Data collection

**Figure 1:**
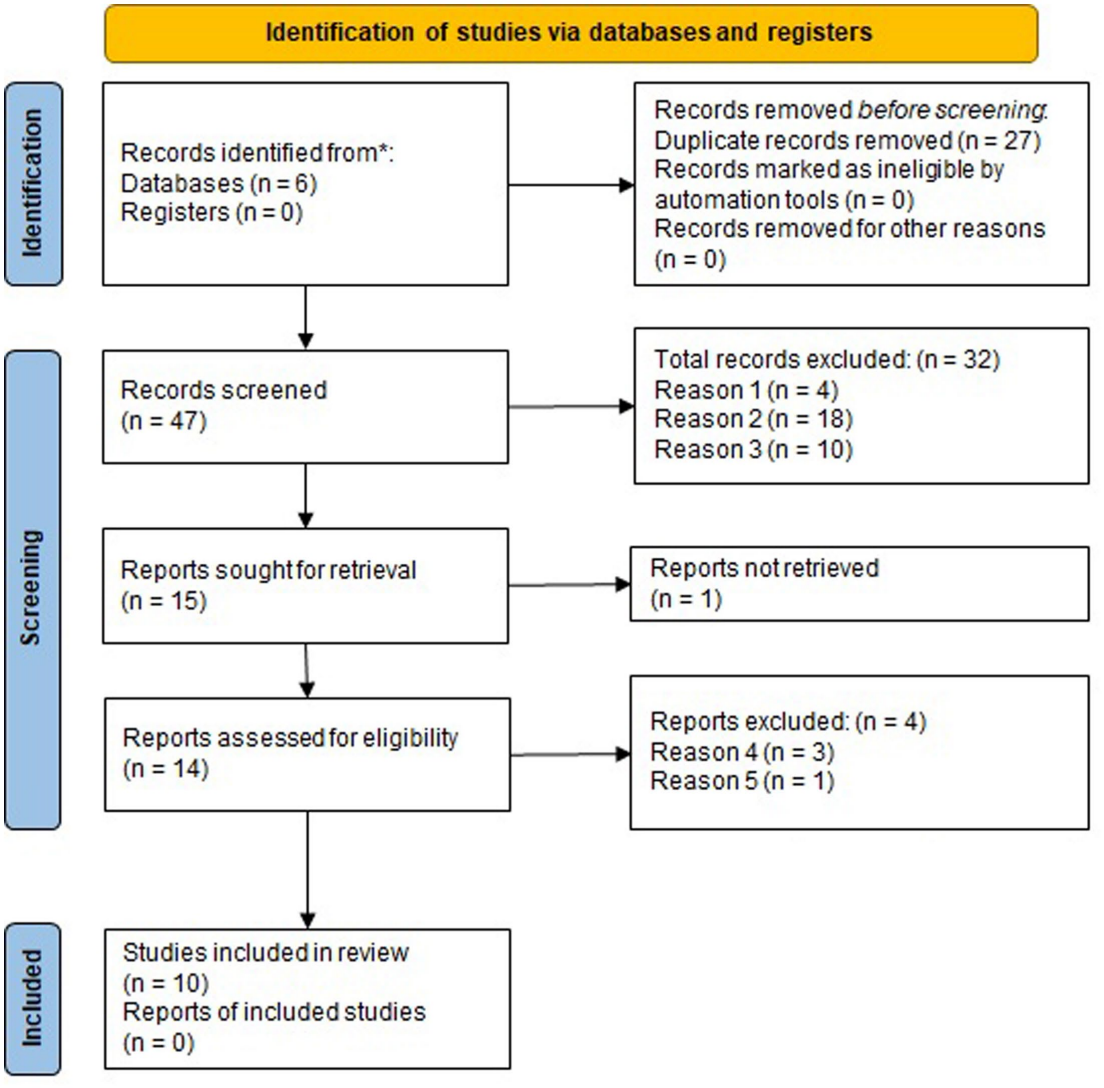
PRISMA diagram of meta-analysis: Reason 1: Not treatment-outcome focused case series or meta-analysis. Reason 2: Single case report. Reason 3: Not about ANE. Reason 4: Did not report individual patient data (treatment and associated outcome). Reason 5: only reported steroid use and did not report timing.

Data collection was performed by author JMF (MS, MD) on 4/4/23. We searched 6 databases (Pubmed, Ovid, Cochrane, CINAHL, Embase and Scopus) using the following input: “(“Acute necrotizing encephalopathy” OR “ANEC”) AND (IVIG OR Steroids OR Plasma exchange OR Tocilizumab OR neuroimmunology) AND (outcomes OR neurologic outcome OR neurological sequelae)” between the years 2003 and 2023 (Figure 1). None of the articles screened were in a primary language other than English. Our search algorithm did not include abstracts or unpublished studies and therefore none were identified for screening. Forty seven unique articles were identified for screening. We manually screened the title and abstract and 32 records were excluded for at least one of the following reasons: (1) Not treatment/outcome focused; (2) Single case report; (3) Not about ANE. Fourteen of the 15 articles sought for retrieval were able to be retrieved. Out of the 14 articles assessed for eligibility, 4 were excluded due to at least one of the following reasons: (4) did not report individual patient data or (5) only immunosuppressive therapy was steroids, but did not report timing. High-dose steroids was defined as IV methylprednisolone at a dose of at least 30 mg/kg/day (8). Ten articles, with a total of 158 patients were included in the meta-analysis (Table 1).

**Table 1:** Detailed description of included journal articles in meta-analysis.

	Author/paper details	Article name	Analysis	Outcome	RANBP2?	N	Good outcome
1	Okumura et al. (14) Japan	Outcome of acute necrotizing encephalopathy in relation to treatment with corticosteroids and gammaglobulin	Steroids (1), IVIG (2)	Good–DQ/IQ ≥ 50 Poor–DQ/IQ < 50	Not Reported	17	7/17 (41%)
2	Lim et al. (12) Singapore	Serial outcomes in acute necrotizing encephalopathy of childhood: A medium-and long-term study	Steroids (1), IVIG (2), Combination (3)	Good-PCPC 1–3 Poor-PCPC 4–6	Not Reported	7	3/7 (43%)
3	Koh et al. (20) Singapore	Favorable Outcomes With Early Interleukin 6 Receptor Blockade in Severe Acute Necrotizing Encephalopathy of Childhood	Steroids (1), IVIG (2), Combination (3), Tocilizumab (4)	Good-mRS 0–2 Poor-mRS 3–6	No	3	3/3 (100%)
4	Appavu et al. (8) USA	Treatment Timing, EEG, Neuroimaging, and Outcomes After Acute Necrotizing Encephalopathy in Children	Steroids (1), IVIG (2), Combination (3), Tocilizumab (4)	Good-mRS 0–2 Poor–mRS 3–6	No	7	2/7 (29%)
5	Askoy et al. (22) Turkey	Acute necrotizing encephalopathy of childhood: A single-center experience	Steroids (1), IVIG (2), Combination (3), PLEX (5)	Good-no or “mild” sequelae Poor-“moderate,” “severe” or death	No	9	2/9 (22%)
6	Zhu et al. (10) China	The Clinical and Imaging Characteristics Associated with Neurological Sequelae of Pediatric Patients with Acute Necrotizing Encephalopathy	Steroids (1), IVIG (2)	Good– “mild” DQ/IQ ≥ 50 Poor– “moderate,” “severe” DQ/IQ < 50 or death	Not Reported	34	8/34 (24%)
7	Chang et al. (11) Taiwan	Early High-Dose Methylprednisolone Therapy Is Associated with Better Outcomes in Children with Acute Necrotizing Encephalopathy	Steroids (1), IVIG (2), Combination (3)	Good–PCPS 1–3 Poor–PCPS 4–6	Not Reported	26	10/26 (38%)
8	Lee et al. (23) Malaysia	Factors associated with outcomes of severe acute necrotizing encephalopathy: A multicenter experience in Malaysia	IVIG (2), Tocilizumab (4)	Good-mRS 0–2 Poor–mRS 3–6	No	24	6/24 (25%)
9	Okajima et al. (19) Japan	Early therapeutic plasma exchange may lead to complete neurological recovery in moderate to severe influenza-associated acute necrotizing encephalopathy	PLEX (5)	Surival during hospitalization	Not Reported	2	Survival: 2/2 (100%)
10	Li et al. (18) China	Plasma exchange therapy for acute necrotizing encephalopathy of childhood	PLEX (5)	Survival during hospitalization	Not reported	29	Survival: 10/29 (34%)
					Total # patients	158	53/158 (34%)

BS, brainstem involvement; PCPS, Pediatric Cerebral Performance Scale; PCPC, Pediatric overall performance category; DQ/IQ, Gesell Developmental scales or Wechsler Intelligence Test; Steroids, pulse dose intravenous solumedrol unless otherwise specified.

### 2.3. Data analysis

Data from each paper was extracted with respect to the following variables: treatment, outcome, and brainstem involvement. Five independent analyses were performed, each designed to answer specific clinical questions about immunosuppressive treatment. The first evaluated the benefit of early treatment with steroids (ES). ES was defined as high dose steroid treatment with intravenous methylprednisolone (IVMP) initiated within 24 h of neurologic decline, compared to late treatment termed (LS)—high dose IVMP initiated later than 24 h after neurologic decline. In this analysis we divided patients into three main groups: ES, LS and no steroids (NS), and two combination groups: any steroids (AS) which included both ES and LS, and no early steroids (nES) which included patients who received LS or NS. The time frame of 24 h was chosen in order to be consistent with a prior report demonstrating benefits of ES (11). Articles were not included in this analysis if they did not definitively report the timing of steroid treatment.

The second compared outcomes in patients treated with or without the IVIG, independent of other treatments. Articles were not included in this analysis if they did not report the use of IVIG.

The third investigated the benefit of combination therapy with early steroids and IVIG. In this analysis patients were divided into three groups: those treated with ES and IVIG, ES alone, and neither ES nor IVIG. We did not include a group with IVIG alone because the sample size in this condition was small and as our prior analysis did not demonstrate a benefit to IVIG alone this was not felt to be a valuable subcategory.

In the fourth analysis, we investigated the benefit of the IL-6 inhibiting monoclonal antibody, tocilizumab. Articles were not included in this analysis if they did not report timing of steroid treatment, did not use both tocilizumab and steroids in at least one patient, or if they did not indicate which patients received combination therapy versus those who only received a single modality. Patients were divided by those receiving and those not receiving tocilizumab, as well as those receiving a combination of tocilizumab and ES, ES alone or neither therapy.

Last, for our fifth analysis we identified three papers which evaluated the effectiveness of plasmapheresis (PLEX) in patients with ANE. Only one of these papers included morbidity outcomes, but all three reported mortalities during hospitalization, thus this outcome was analyzed instead. Articles were only included for this analysis if at least one patient received PLEX.

Criteria used to determine good vs. poor outcome was reported differently in many of the papers. To allow for pooled analysis, outcomes were divided into “good” and “poor” using as similar criteria as possible (Table 1). For the steroids and IVIG analyses, a sub-group analysis of patients with and without brainstem (B/S) involvement was performed; if an article did not report the presence or lack of brainstem involvement, these patients were included in the “all” category, but not included in the sub-group analysis. It was not possible to use all 10 articles for all five analyses; Table 1 indicates which articles were used for which analyses.

In all analyses (other than PLEX), the quantity of patients reported to have a “good outcome” were divided by the total number patients in that category to calculate the % good outcome. For the PLEX analysis, % survival was calculated instead. Frequencies of good vs. bad outcomes (or death for PLEX analysis) were compared individually with each other group and analyzed statistically using either 2×2 Chi-squared tests when all groups had an expected count >5 or Fisher’s exact testing when at least one group had an expected count <5. Both statistical tests were performed using a *Social Science Statistic (Washington, DC, United States)* online calculator. For all analyses where multiple comparisons were performed on the same dataset, a Bonferroni correction was used to reduce the risk of type 1 error. For the first analysis, the threshold for significance was adjusted to *p* = 0.01 for each group (all, no brainstem and brainstem) to account for each of the five comparisons. No adjustment was necessary for the IVIG analysis. For the combined steroid and IVIG analysis, the threshold for significance was adjusted to *p* = 0.017 to account for the three comparisons. For the tocilizumab analysis, no adjustment was required when comparing tocilizumab vs. no tocilizumab in all comers, however when groups were divided by tocilizumab ± ES, the threshold for significance was adjusted to 0.017 to account for the three comparisons being performed on each dataset. No Bonferroni adjustment to significance was required for the PLEX analysis.

## 3. Case report

**Figure 2:**
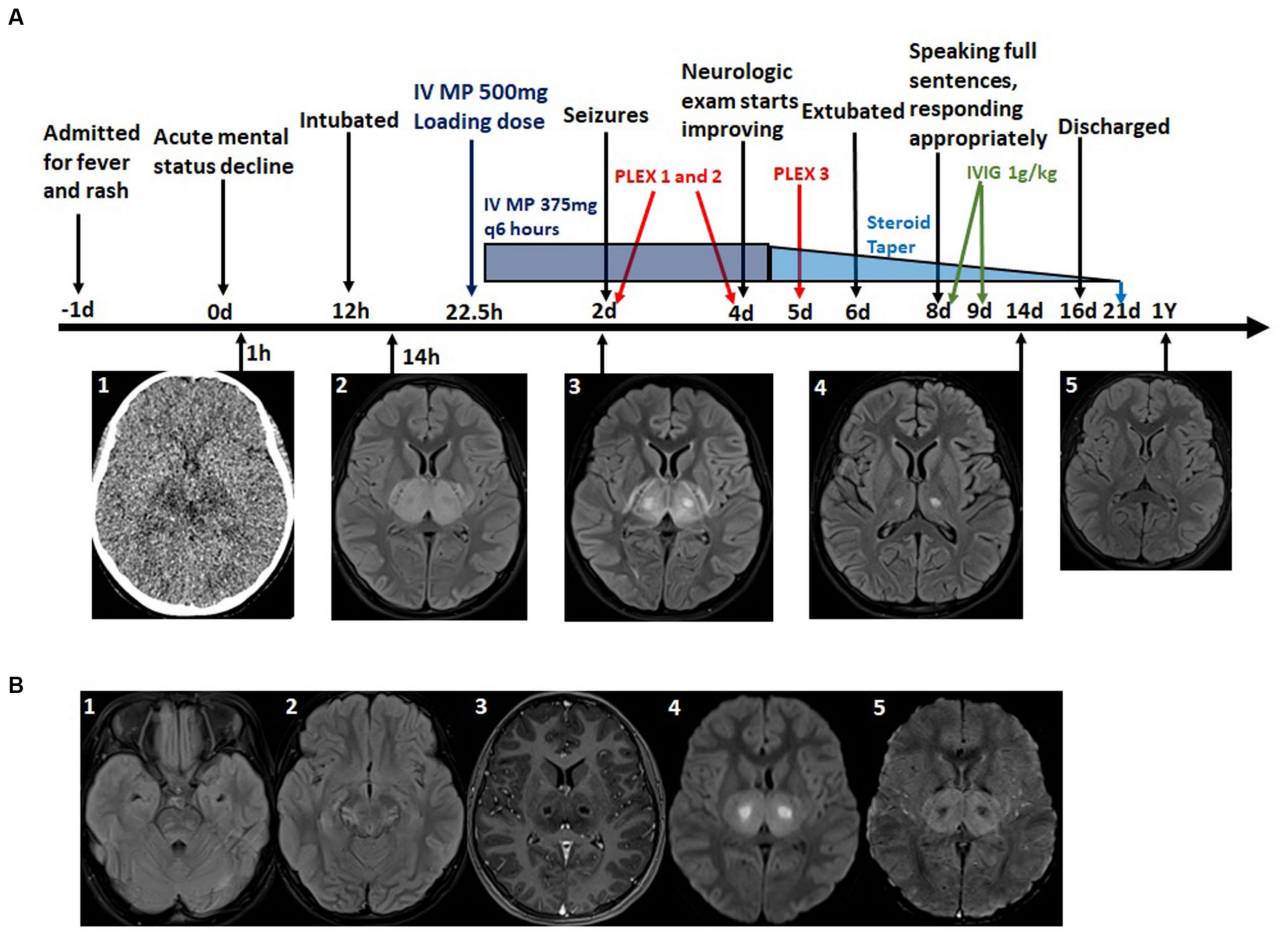
**(A)** Timeline of major clinical events, Imaging findings and immunosuppressive treatments. A1: CT scon at time of mental status change-bilateral thalamic hypodensities. A2–5: T2 Flair MRI at level of thalamus at indicated time points-time course of resolution of bilateral thalamic lesions. **(B)** Additional relevant MRI imaging findings. B1: T2 Flair MRI at time of initial MRI-bilateral pontine hyperintensity, subtle right temporal lobe edema. B2: T2 Flair MRI on day 2 - midbrain edema. B3: T1 with contrast MRI demonstrating ring enhancing thalamic lesions. B4: Diffusion weighted imaging (DWI) MRI-core of thalamic lesions restrict diffusion. B5: Susceptibility weighted imaging (SWI) MRI hemorrhage in thalamic lesions.

A 10-year-old previously healthy female first presented to an outside hospital with fever, malaise, and abdominal pain. It was presumed to be due to a urinary tract infection and she was discharged home on amoxicillin. Two days later, she returned to the same emergency department for persistent fevers. Labs revealed neutropenia, elevated transaminases, elevated D-dimer and lactate dehydrogenase and viral testing, including COVID-19, was negative (Table 2). She was again discharged with a longer course of different antibiotics. While at home she continued to be febrile and developed worsening abdominal pain and new diarrhea. Several days later, now 3 weeks from the onset of her symptoms, she developed higher fevers and a new rash, which prompted return to the emergency department. At that time, her exam was notable for hepatomegaly and a diffuse erythematous blanching macular rash. She had worsening leukopenia but down-trending transaminases (Table 2). She was admitted to the acute care floor where cultures were obtained, and IV antibiotics were initiated. It is noteworthy that, while she had a multitude of other systemic symptoms, her mental status remained intact until the first night of admission, per report of her parents. On the night of admission, she acutely developed diplopia, hallucinations, and progressive lethargy over several hours with head computed tomography (CT) demonstrating symmetric hypodensities in the bilateral thalami (Figure 2).

**Table 2:** Relevant serum and CSF studies in our patient.

Serum	ED Visit (3 weeks prior to ANE)	Admission (Day 0 of ANE)	PICU (Day 1 of ANE)	Infectious		Immunologic	
WBC (K/mcL)	3.8	2.9		Respiratory Viral PCR*	Negative	Autoimmune Encephalitis Antibody Panel****	Negative
Lymphocytes %	58	58.6	5	SARS-COV-2 PCR	Negative	Antinuclear antibody	WNL
Hemoglobin (g/dL)	12.6	12.6	9.4	Bartonella IgG and IgM (Henselae and Quintana)	Negative	C3 Complement	WNL
Platelets (K/mcL)	208	179	90	CMV IgG, IgM, and DNA Quant	Negative	C4 Complement	WNL
Na (mmol/L)	138	137	143	SARS-COV-2 Antibodies	Negative	IgG	WNL
Bicarbonate (mmol/L)	24	21	17	EBV IgG	Elevated	IgA	Elevated
BUN (mg/dL)	10	11	10	EBV IgM, PCR	Negative	IgM	WNL
Cr (mg/dL)	0.6	0.62	0.63	HHV-6 PCR	Negative	IgE	WNL
Glucose (mg/dL)	95	102	100	HSV 1 and 2 PCR	Negative	Typhus Fever IgG and IgM	Negative
AST (units/L)	99	54	289	Influenza A and B AG	Negative	Tickborne Panel PCR**	Negative
ALT (units/L)	194	54	183	Leptospira IgM	Negative	Arbovirus Antibodies IgM and IgG***	Negative
LDH (units/L)	871	871	–	Lyme DNA PCR	Negative	Aquaporin-4 Antibody	Negative
CRP (mg/dL)	1.2	<0.5	5.1	Monospot	Negative		
D-Dimer (ng/ml FEU)	510	920	–	Mycoplasma Pneumonia IgG/M	Negative		
CSF	Basic			Infectious/Autoimmune			
Glucose (mg/dL)	59			HSV 1 and 2 IgM		Negative	
				Lyme Antibody		Negative	
Protein (mg/dL)	105			Arbovirus Antibodies IgM and IgG***		Negative	
RBC (RBC/mcL)	29			Meningitis/Encephalitis panel PCR*****		Negative	
WBC (WBC/mcL)	1			Genetics	RANBP2	Negative	

WBC, white blood cell count; RBC, red blood cell count; K, thousand cells; mcL, microliters; g, gram; dL, deciliter; Na, sodium; mmol, millimoles; L, Liter; BUN, blood urea nitrogen; mg, milligram; Cr, Creatinine; g, gram; AST, aspartate aminotransferase, ALT, alanine aminotransferase; Alk Phos, alkaline phosphatase; CRP, C-reactive protein; ng, nanogram; mL, milliliter; CSF, cerebrospinal fluid, PCR, polymerase chain reaction; SARS-COV-2, severe acute respiratory syndrome-coronavirus disease 2019; IgG, Immunoglobulin G; IgM, Immunoglobulin M; CMV, cytomegalovirus; DNA, deoxyribonucleic acid; Quant, Quantitative; EBV, Epstein–Barr virus; IgA, Immunoglobulin A; IgE, Immunoglobulin E; HSV, Herpes simplex virus; HHV-6, human herpes virus 6; WNL, within normal limits; RANBP2, RAN binding protein 2.

*Adenovirus, influenza A&B, Human metapneumovirus, parainfluenza, coronavirus, RSV, rhino/enterovirus, mycoplasma pneumonia, *Bordetella pertussis* and parapertussis, and chlamydia pneumonia.

**Anaplasma phagocytophilum, *Ehrlichia chaffeensis*, *Ehrlichia ewingii/canis*, *Ehrlichia muris*-like.

***St. Louis, California, Eastern Equine, Western, West Nile.

****Includes N-methyl-D-aspartate receptor, Voltage-Gated Potassium Channel, Glutamic Acid Decarboxylase, Aquaporin-4 Receptor antibodies.

*****Includes *Escherichia coli*, *Haemophilus influenzae*, *Listeria monocytogenes*, *Neisseria meningitidis*, *Streptococcus agalactiae*, *Streptococcus pneumoniae*, Cytomegalovirus, Enterovirus, Herpes simplex virus 1, Herpes simplex virus 2, Human herpesvirus 6, Human parechovirus, Varicella zoster virus, *Cryptococcus neoformans*/*gatt*.

She was transferred to the pediatric intensive care unit (PICU) where she was noted to be confused, agitated, unable to follow commands, and had bowel and bladder incontinence. Her mental status waxed and waned; at worst, she was unresponsive. Hyperreflexia in the bilateral upper and lower extremities were appreciated on exam. Given her mental status, she was endotracheally intubated for airway protection. Antimicrobials were broadened for meningitis and encephalitis coverage. MRI brain, with and without contrast, showed symmetric, bi-thalamic T2 hyperintensity with central oval shaped cystic areas with rim-enhancement, faint hemorrhage, circumferential diffusion restriction, and multifocal T2 hyperintense lesions, consistent with vasogenic edema, in the bilateral cortex, juxtacortical white matter, bilateral hippocampi, midbrain, pons and cerebellum (Figure 2). MRI of the cervical, thoracic, and lumbar spinal cord was not performed at time of the initial diagnosis; however, it was performed prior to discharge and no abnormalities were identified. A diagnosis of ANE was made based on characteristic MRI findings and clinical presentation. An extensive workup for alternative or additional etiologies was completed and ultimately negative (Table 2). Lumbar puncture revealed a cerebrospinal fluid (CSF) protein of 105 (normal <60) (Table 2). Many additional tests were performed and are detailed in Table 2. These tests included genetic testing for RANBP2, and Aquaporin-4 antibodies were both negative. Unfortunately, oligoclonal bands and myelin oligodendrocyte glycoprotein (MOG) antibodies were not sent. Because of fluctuations in level of awareness, she started on continuous electroencephalography (EEG) which showed right sided temporal lobe seizures. She was loaded with fosphenytoin 20 mg/kg resulting in resolution of her seizures. She continued fosphenytoin 100 mg twice per day for maintenance therapy which was transitioned to oral levetiracetam 500 mg twice per day after 10 days. ANE-SS was calculated as 6/9 due to older age, low platelet count, presence of elevated csf protein and presence of brainstem involvement on imaging (Table 3).

**Table 3:** ANE illness severity score (17) calculation in our patient.

ANE-SS criteria	Possible points	Our patient
Shock	3	No–0
Age > 48 months	2	Yes–2
Brainstem involvement	2	Yes–2
Platelet count <100,000	1	Yes–1
CSF protein <60 mg/dL	1	Yes–1
Total	9	6

She was given a loading dose of IV MP 500 mg 22.5 h after acute mental status decline (Figure 2) and continued IV MP 375 mg every 6 h (total daily dose 30 mg/kg) for four more days. Followed by a 14-day oral steroid taper with prednisone. She received three rounds of PLEX, 1.5 times volume, on days 2, 4, and 5 (originally planned for five rounds, but stopped early due to coagulopathy). Finally, she was given IVIG, 1 g/kg, on days 8 and 9. Because of the association of ANE with impaired cellular metabolism, she was also given a mitochondrial cocktail of nutritional supplements consisting of—CoQ10 2–8 mg/kg/day, Riboflavin 100 mg per day, L-carnitine 100 mg/kg/day, Leucovorin 0.5–2.5 mg/kg per day, Thiamine 100 mg per day, and Biotin 5–10 mg per day. Additional neuroprotective strategies were employed throughout her PICU course, including elevating the head of the bed and maintaining normal oxygenation, normocarbia, normothermia, normotension, normoglycemia, and normonatremia.

After 24 h of high dose steroids, her mental status improved with the ability to follow commands. Antimicrobials were discontinued when cultures and infectious studies were negative at 48 h. Four days following PICU admission, she was extubated. Upon transfer to the pediatric floor, she was alert and oriented with fluent speech and no cranial nerve deficits. She had generalized weakness, but was able to ambulate short distances without assistance. Repeat brain MRI 14 days after her initial study demonstrated marked improvement of previously noted edema with minimal residual abnormal T2 signal in the bilateral thalami, inferior and high frontal cortex (Figure 2).

She was discharged home on hospital day 18 with intensive outpatient therapy, including attending a therapeutic school at a local rehabilitation facility. Testing revealed a drop from above average to average academic performance, requiring supportive accommodation at school. She remained seizure free and levetiracetam was weaned off 6 months after discharge without issue. Her mitochondrial cocktail was discontinued 1-month post-discharge except for Leucovorin which was discontinued 6 months post-discharge. At her 3-year follow up appointment, she had not had any neurologic worsening or new neurologic symptoms. She was back in her regular school, on the Honor Roll. Her only remaining symptom is a mild postural tremor in her left hand. Her parents are pleased with her progress.

## 4. Results

**Figure 3:**
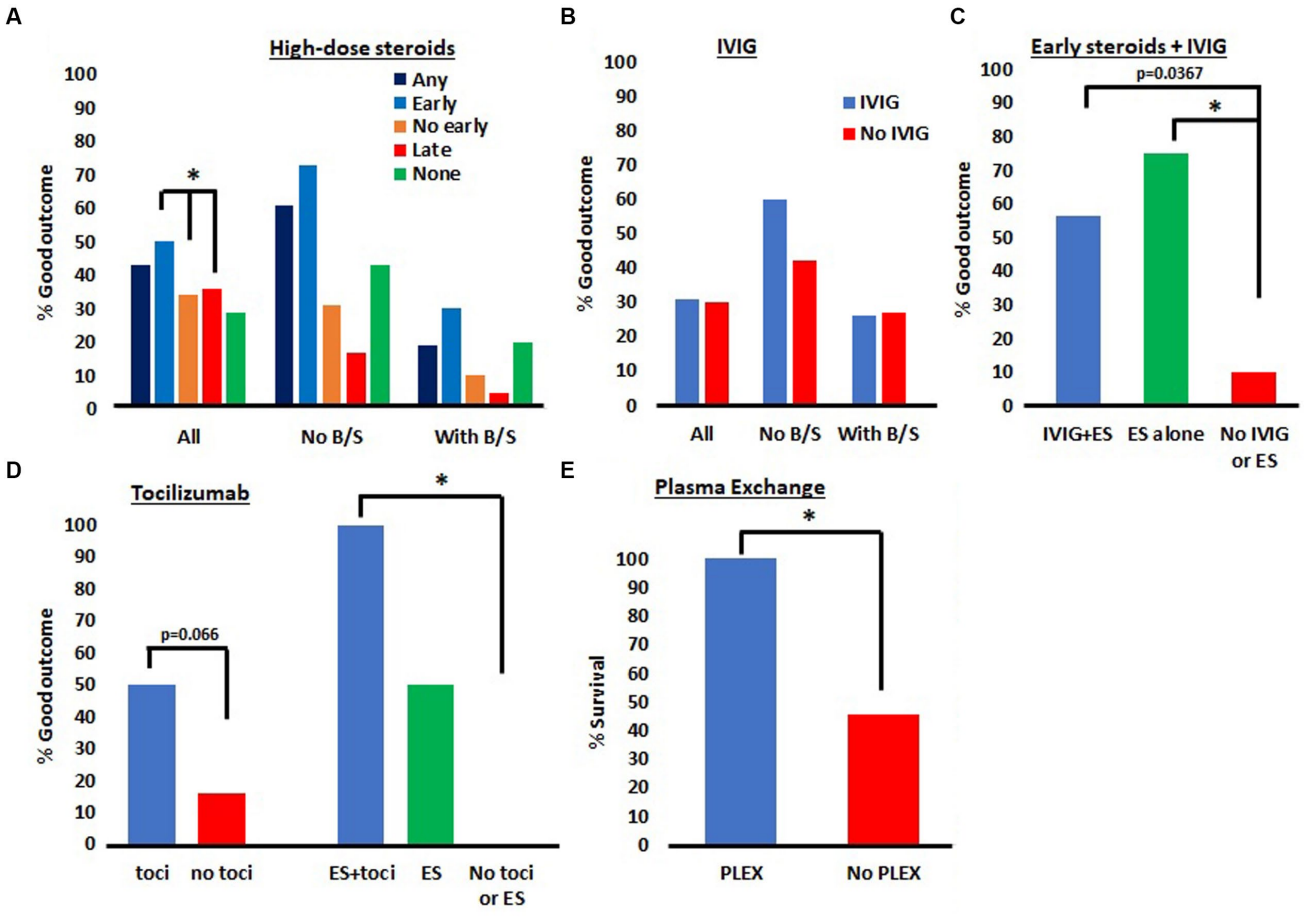
Meta-analysis of ANE outcomes in patients treated with different immunosuppressive agents. **(A)** Outcomes of patients treated with early, late or no IV steroids. Patients treated with early steroids had better outcomes than those treated without early steroids or with late steroids. *p < 0.01. **(B)** There were no differences observed in patients treated with IVIG. **(C)** Combination therapy with IVIG and ES did not lead to superior outcomes than ES alone. *p < 0.017. **(D)** There was a trend toward a significant improvement in patients treated with tocilizumab and a significant Improvement in patients treated with a combination of ES and tocilizumab. *p < 0.017. **(E)** There was a significantly greater % of patients who survived after treatment with PLEX compared to no PLEX. *p < 0.05.

We first examined the outcomes of patients based on steroid treatment and timing (Figure 3A). This analysis was performed irrespective of treatment with IVIG or other immunosuppressive therapies. As stated in methods, patients were divided into five groups—ES, LS, NS, AS, and nES. Eight case series were included in this analysis (8, 10–12, 14, 20, 22, 23). In the first subset of this analysis all patients were considered. We then subdivided our groups by the presence or absence of B/S involvement (using only papers which reported this consistently). In the group of all patients, the proportion of patients in each group with a good outcome were: ES 24/50 (48%); LS 6/47 (13%); NS 5/17 (29%); AS 30/97 (31%); and nES 11/64 (17%). Chi-squared testing was used with Bonferroni correction to a significance threshold to 0.01. The only significant comparisons of any groups were ES vs. LS (*p* = 0.0002) and ES vs. nES (*p* = 0.0004). We then evaluated patients without B/S involvement. The proportions of good outcome in each group were: ES 16/22 (73%); LS 1/5 (20%); NS 3/7 (43%); AS 17/27 (63%); and nES 4/12 (33%). Fisher’s exact testing was used to compare each of these five groups and no significant differences were identified after adjusting the threshold of significance to *p* = 0.01 for Bonferroni correction. In patients with B/S involvement, the proportions of good outcome were: ES 7/23 (30%); LS 2/19 (11%); NS 2/10 (20%); AS 9/42 (21%); and nES 4/29 (14%). Groups in this sample had a larger sample size and Chi-square testing was used to compare each of these groups. No significant differences were identified.

We then evaluated the outcomes in patients treated with or without IVIG, irrespective of steroid treatment (Figure 3B). Eight case series were included in this analysis (8, 10–12, 14, 20, 22, 23). First, we analyzed all patients, regardless of B/S involvement. In this analysis, of those who received IVIG, 28/89 (31%) had good outcomes, while those in who did not receive IVIG 16/54 (30%) had good outcomes. Chi-square test confirmed this difference was not significant. In patients without B/S involvement, 9/15 (60%) had good outcomes with IVIG while 8/19 (42%) had good outcomes without IVIG. Though there was a greater percentage of good outcomes in the IVIG group, this difference was not significant on Chi-square testing. Finally, in those with B/S involvement, 7/27 (26%) had good outcomes with IVIG while 8/30 (27%) had good outcomes without it. Chi-square test confirmed this difference was not significant.

We then sought to evaluate combination therapy with ES and IVIG compared to either therapy alone or no therapy (Figure 3C). Five papers were included in this analysis (8, 11, 12, 20, 22) resulting in a smaller sample size than the prior analyses. Because the sample size was smaller, we were not able to subdivide groups by B/S involvement. The proportion of patients with a good outcome after receiving both ES and IVIG was 9/16 (56%), ES alone was 6/8 (75%), and in those receiving neither therapy it was 1/10 (10%). Fisher’s exact testing with Bonferroni correction to a significance threshold of 0.017 was used for statistical analysis. When ES + IVIG was compared to neither therapy the value of p was 0.0367 which did not meet our threshold for significance after Bonferroni correction. When ES was compared to neither therapy, *p* = 0.012, which was significant. There was no significant difference between ES + IVIG and ES alone.

Our next analysis aimed to assess outcomes associated with or without the use of tocilizumab (Figure 3D). This analysis included three papers (8, 20, 23). In the tocilizumab group, 7/14 (50%) had good outcomes while in the no tocilizumab group 4/20 (20%) had good outcomes. Chi-squared test revealed a *p*-value of 0.066, which was not statistically significant though did trend toward significance. We then looked at combination therapy with ES + tocilizumab vs. ES alone vs. no tocilizumab or ES. The % good outcome with ES and tocilizumab was 5/5 (100%), ES alone was 1/2 (50%) and neither tocilizumab nor ES was 0/4 (0%). Fisher’s exact testing with a Bonferroni correction to a level of significance of 0.017 was used to compare each group. The difference between ES + tocilizumab and neither therapy was found to be significant with a *p*-value of 0.008. No other significant differences were identified.

Last, we wondered if PLEX were associated with superior outcomes in patients with ANE. We identified three articles in which PLEX was used for the treatment of ANE (18, 19, 22). In the group receiving PLEX, 18/18 (100%) patients survived compared to the no PLEX group in which only 12/22 (45%) of patients. Fisher’s exact test identified a significant difference between these groups with a *p*-value of 0.0001.

## 5. Discussion

In this report, we discuss a case of a young girl with clinical-radiographic findings consistent with ANE and an ANE-SS of 6, suggesting severe disease. She received aggressive immunosuppressive therapies detailed in Figure 2, including early HD-IV-MP, IVIG, and PLEX. She ultimately had a good outcome and is now doing well at home and in school. We followed up this case report with a systematic meta-analysis which included 10 case series investigating morbidity (or mortality in the case of the PLEX analysis) outcomes in patients with ANE. The goal of our meta-analysis was to provide data from a broader range of clinical settings and large number of patients to investigate various immunosuppressive interventions in the treatment of ANE. We found that the early high dose IV steroids (methylprednisolone) lead to superior outcomes compared to patients who did not receive early steroids and those receiving late steroids specifically. We did not find any benefit of IVIG, both irrespective of other immunosuppressive therapies, or in combination with ES. We found a trend to suggest that tocilizumab may be beneficial in improving morbidity outcomes, which was significant when added to ES. Last, we found that PLEX was associated with increased survival in patients with ANE.

First, some thoughts regarding our case. Our patient presented to an emergency department (ED) three times over a three-week period with a persistent febrile illness, associated with diarrhea, abdominal pain, and elevated liver enzymes. Her third presentation was to an ED at our hospital where she was admitted. On night-one of admission, she had an acute neurologic decline and urgent CT scan of her head revealed bilateral thalamic hypodensities. This was followed up with an MRI which was consistent with ANE with brainstem involvement. She received a thorough evaluation for additional or alternative pathologies associated with her presentation, but all tests were negative. A respiratory viral panel including influenza, as well as extensive testing for other viruses was obtained and negative. It remains unclear what the inciting infectious trigger for her presentation was and it is noteworthy that that 3 weeks of viral illness and liver injury are highly atypical for a respiratory viral illness which is the most frequent trigger of ANE. Regardless, imaging strongly suggested ANE with brainstem involvement and an ANE severity score was 6/9, which is considered high risk for a poor outcome (17). Of the 17 patients in the high risk category noted in Yamamoto et al., 15 had an outcome categorized as “severe sequelae or death” (17). It was very fortunate that our patient survived with minimal sequelae and is now back at school living a life like her pre-illness state. While it is not possible to know all the factors that enabled our patient’s successful recovery, it is likely that at least some of our immunosuppressive treatments (ES, PLEX and IVIG) contributed to her good outcome. While many have published case series investigating immunosuppressive treatment strategies in patients with ANE, we were not able to find a unified, evidence-based treatment algorithm to guide management of these patients. The studies we encountered were all from a single center, in a single geographic region, thus external validity was limited. Because sample sizes were relatively low, it was not always possible to draw statistically significant conclusions from these reports. For these reasons, we aimed to add to this body of literature by conducting the first systematic meta-analysis of immunosuppressive therapies and associated outcomes for patients with ANE.

By combining the data from multiple centers, we hoped to mitigate biases inherent in studies originating from a single center. That said, the homogenization of data obtained from multiple sources also presents its own set of limitations. First, some of the case series provided a much larger sample than other case series, thus our data was skewed to favor the results of the larger case series. Because medical resources and medical practices differ greatly between different centers and different countries, outcomes also differ, irrespective of immunosuppressive therapy. For this reason, data from centers that more closely match one’s own center may be more valuable than composite data from many centers. Unfortunately, since ANE is so rare and sample sizes are limited, it may not always be possible to draw statistically significant conclusions from a single center or single region.

Next, the diagnosis of ANE is based on clinical and radiologic findings, however underlying pathogenesis is variable. Subtypes of ANE, such as those caused by RANBP2 mutations are likely to respond differently to therapies. While most of the papers included in our study reported that none of their patients were RANBP2 positive, several papers did not include this detail thus it is probable that our meta-analysis includes at least some patients of this alternative sub-type. Because no papers in our analysis reported RANBP2 positive patients, we feel our results are more applicable to those without RANBP2 mutations. Additional inquiry would be required to evaluate treatment efficacy in RANBP2 positive patients.

In our analysis, when possible, we chose to divide patients into those with and without B/S involvement as a marker of disease severity (those with B/S involvement being more severe). This factor was chosen, rather than the ANE-SS, because it was reported more frequently in the included studies. However, the ANE-SS is a validated as a marker of ANE severity while B/S involvement alone is not, though B/S involvement is included in this score. Our analysis did not identify any significant difference between patients with and without B/S involvement which may be because B/S involvement does not impact ANE treatment, or it may be because our sample sizes were smaller in the subgroups divided by B/S involvement. Regardless, since the ANE-SS is validated, we suggest using the ANE-SS rather than B/S involvement alone to guide treatment decisions. The study first describing the ANE-SS divided patients into three categories based on ANE-SS (low-risk 0–1; medium-risk 2–4; and high-risk 5–9) (17). In their study, the incidence of severe sequelae or death was 27.3% in the low-risk group, 66.7% in the medium-risk group and 88.2% in the high-risk group (17). Given the substantial morbidity and mortality in both the medium and high-risk groups we suggest using a ANE-SS of 2 as a cutoff for more aggressive therapy with tocilizumab and/or PLEX. In doing so, we suggest this more aggressive approach to treatment until additional data becomes available.

Another limitation of our analysis is that we only used repeated univariate statistical analyses (Chi-square and Fisher’s exact testing), rather than multivariate analysis. This was done to simplify our analysis and to reduce costs, as our work was not externally funded. That said, multivariate analyses would have allowed us to compare a treatment specific treatment effect compared to all other groups simultaneously instead comparing to each other treatment individually within the same dataset. To reduce the risk of the bias generated by our statistical methodology, we applied a Bonferroni correction, adjusting the value of p threshold of significance based on the number of comparisons performed within each group.

One challenge we faced when performing our analysis was that morbidity outcomes were reported using several different metrics. To include as many studies as reasonably possible, improving the power of our analysis, we pooled outcome data into two categories of “good” and “poor.” While this method increased heterogeneity of patients considered to have a “good” outcome, we trusted that each study chose a metric which was representative of a good outcome in their patient population with their level of health care resources. For this reason, we hoped that the stringency of their chosen metric would control differences in health care quality and improve our ability to examine the effect of immunosuppressive therapy. While we recognize this also as a limitation, we hope that our analysis will provide useful information for future clinicians and patients faced with this challenging disease.

Others have demonstrated, in single centers, that ES (i.e., pulse dose steroids initiated within 24 h of onset of neurologic symptoms), but not late steroids, are associated with better outcomes in patients with ANE (11, 14). This finding was confirmed in our larger sample size spanning 127 patients from 8 different clinical centers in the steroid treatment analysis. This finding was not replicated in either patients with or without B/S involvement. The fact that it was not replicated in either subgroup suggests that the sample sizes were too small to identify this difference in at least one of the conditions. It is also worth noting that LS treatment did not provide any benefit and was even found to have a lower proportion of patients with a good outcome compared to no steroid treatment at all. This strongly reinforces the need for early recognition of ANE and urgent initiation of high dose IVMP without delay. The mechanism by which timing is so important in steroid treatment of ANE remains unclear, however, it stands to reason that later phases of the disease process are not impeded greatly by steroids. Lastly, our study was not able to stratify patients in the LS group to different time points, for this reason, the time frame when steroids become ineffective is not yet known. Until this threshold can be identified, it is likely worthwhile to treat all patients with high dose IV steroids as the risks are relatively small.

We did not find any benefit in patients treated with IVIG compared to those who were not in all patients, patients without B/S involvement or patients with B/S involvement. Consistently, patients treated with IVIG and ES did not have better outcomes than those treated with ES alone. For these reasons, we do not feel that IVIG is an effective tool in the management of ANE.

We also analyzed outcomes of ANE with and without treatment with an IL-6 inhibitor, tocilizumab. Prior investigation supports a central role of the cytokine IL-6 in the pathogenesis of ANE and higher IL-6 levels have been found to correlate with more severe disease (24). IL-6 inhibition is a natural choice when considering a more targeted approach to treating ANE. Unfortunately, our sample size was small and although the proportion of patients found to have a good outcome after receiving tocilizumab was much higher than those not receiving tocilizumab, this finding did not reach statistical significance. It is certainly possible that further evaluation of tocilizumab with a larger sample size would yield significant results. That said, it is also important to acknowledge selection bias in case series focused on a single intervention. It is possible that tocilizumab was preferentially added to a patient’s therapy in more severe cases, resulting in a severity bias. This bias would increase the risk of a false negative (type two error) and thus may have decreased our ability to identify a benefit with tocilizumab. We also looked at combination therapy with both tocilizumab and ES. While we did find a significant increase in the proportion of good outcomes after tocilizumab + ES compared to neither therapy, we were most interested in comparing tocilizumab + ES to ES alone and our sample size was too low in the ES alone group to draw a meaningful conclusion. While the benefit of tocilizumab in ANE is not definitively supported by our analysis, it does suggest a potential benefit and the risks of a short duration of tocilizumab treatment are low. Until more definitive data is reported, it may be worthwhile to use tocilizumab as an adjunctive therapy for most patients with ANE.

In our case report, we decided to prioritize early PLEX on day 2, while she was still receiving high dose steroids. The timing of her neurologic recovery correlated well with the initiation of PLEX. Our meta-analysis identified a significant mortality benefit with the use of PLEX. While we do feel this is a useful finding, it should be interpreted with caution as there are several sources of bias. For one, it may take several days to initiate PLEX thus patients with more severe disease before PLEX can be initiated. This would create bias toward patients with less severe disease receiving PLEX, as they must survive long enough to receive it. There is likely publication bias (studies more likely to be published if they found a therapeutic benefit) and a broader selection bias as patients were not randomly assigned to a PLEX or no PLEX group. Despite these potential biases, the degree of improvement was sizeable. PLEX also carries a higher degree of risks with its use, as it requires the placement of a large bore central line. We feel that PLEX may not be necessary in every patient with ANE, such as those with milder disease which is improving with ES alone. Conversely, our data does support its use to improve mortality and we feel that providers should have a low threshold to use it. We suggest strongly considering PLEX in patients who are worsening despite ES, those who were far out of the window to receive ES, and those with more severe disease (ANE-SS ≥ 2).

In conclusion, we report a case of a patient with high risk ANE, based on ANE-SS, who made a near full recovery. This positive outcome was likely multifactorial, including the early initiation of steroids within 24 h, potentially the use of additional immunosuppressive therapies, and the supportive care she received in our PICU. A standardized treatment algorithm for immunosuppressive therapy in ANE does not yet exist, prompting our meta-analysis of eight case series. Our analysis supported the use of steroids, though especially if initiated within 24 h of presentation. While we do not feel our data is strong enough to develop official treatment guidelines, we do provide several useful insights which may be helpful for clinicians treating ANE. Specifically, our data supports the use of high dose, IV steroids especially when initiated within approximately 24 h of neurologic symptom onset, though the cutoff when steroids no longer add clinical benefit remains undetermined and should be investigated further. In many patients this may be sufficient to offer a high chance of a good outcome. Our analysis does not support the use of IVIG. While our analysis does not strongly support the use of tocilizumab, it does suggest a possible benefit and given the low risk to benefit ratio should also be considered as an adjunctive therapy for many patients. PLEX should be strongly considered, especially for patients with more severe disease or those who did not receive early steroids. We hope that further work investigating immunosuppressive treatments for ANE will add to this knowledge and allow us the best possible chance to help patients with this potentially devastating disease.

## Data availability statement

The original contributions presented in the study are included in the article/Supplementary material, further inquiries can be directed to the corresponding author.

## Ethics statement

Written informed consent was obtained from the legal guardian for the publication of any potentially identifiable images or data included in this article.

## Author contributions

SF, JF, and MR conceptualized the report, completed background literature review, drafted the initial manuscript, and reviewed and revised the manuscript. SF, JF, and TK drafted the tables, figures, and legends, completed background literature review to inform the discussion, reviewed, rewrote portions, and critically revised the manuscript. JT and JF drafted the abstract, gathered information for the tables and figures, reviewed, rewrote portions, and critically revised the manuscript. ST and JF drafted components of the discussion, revised the manuscript, and critically reviewed the manuscript, figure and tables for important intellectual content including imaging. All authors approved the final manuscript as submitted and agreed to be accountable for all aspects of the work.

## Conflict of interest

The authors declare that the research was conducted in the absence of any commercial or financial relationships that could be construed as a potential conflict of interest.

## Publisher’s note

All claims expressed in this article are solely those of the authors and do not necessarily represent those of their affiliated organizations, or those of the publisher, the editors and the reviewers. Any product that may be evaluated in this article, or claim that may be made by its manufacturer, is not guaranteed or endorsed by the publisher.
