# Supplementary material for: Case report: Acute necrotizing encephalopathy: a report of a favorable outcome and systematic meta-analysis of outcomes with different immunosuppressive therapies
Source: Front Neurol. 2023 Sep 1;14:1239746. doi: 10.3389/fneur.2023.1239746 (PMC10512083; doi:10.3389/fneur.2023.1239746)
Supplement: Supplementary file 1 [file Data_Sheet_1.PDF]

Data-base	Citation	Inclu-ded?	Reas-on	Duplic-ate?
Pubmed	Levine JM, Ahsan N, Ho E, Santoro JD. Genetic Acute Necrotizing Encephalopathy Associated with RANBP2: Clinical and Therapeutic Implications in Pediatrics. Mult Scler Relat Disord. 2020 Aug;43:102194. doi: 10.1016/j.msard.2020.102194. Epub 2020 May 15. PMID: 32426208; PMCID: PMC7228726.	No	1	-
Pubmed	Ahmad Malik J, Ahmed S, Shinde M, Almermesh MHS, Alghamdi S, Hussain A, Anwar S. The Impact of COVID-19 On Comorbidities: A Review Of Recent Updates For Combating It. Saudi J Biol Sci. 2022 May;29(5):3586-3599. doi: 10.1016/j.sjbs.2022.02.006. Epub 2022 Feb 10. PMID: 35165505; PMCID: PMC8828435.	No	1	-
Pubmed	Hernandez R, Ota R, Medina Y, Hernandez Y, Julayanont P, Wilms H. Acute necrotizing encephalopathy. Proc (Bayl Univ Med Cent). 2022 Jun 28;35(5):725-727. doi: 10.1080/08998280.2022.2086781. PMID: 35991737; PMCID: PMC9373783.	No	2	
Pubmed	Ndondo AP, Eley B, Wilmshurst JM, Kakooza-Mwesige A, Giannoccaro MP, Willison HJ, Cruz PMR, Heckmann JM, Bateman K, Vincent A. Post-Infectious Autoimmunity in the Central (CNS) and Peripheral (PNS) Nervous Systems: An African Perspective. Front Immunol. 2022 Mar 9;13:833548. doi: 10.3389/fimmu.2022.833548. PMID: 35356001; PMCID: PMC8959857.	No	3	-
Pubmed	Appavu B, Foldes S, Fox J, Shetty S, Oh A, Bassal F, Marku I, Mangum T, Boerwinkle V, Neilson D, Kruer M. Treatment Timing, EEG, Neuroimaging, and Outcomes After Acute Necrotizing Encephalopathy in Children. J Child Neurol. 2021 Jun;36(7):517-524. doi: 10.1177/0883073820984063. Epub 2021 Jan 4. PMID: 33393838.	Yes	-	-
Pubmed	Cleuziou P, Renaldo F, Renolleau S, Javouhey E, Tissieres P, Léger PL, Bergounioux J, Desguerre I, Dauger S, Levy M; Groupe Francophone de Réanimation et Urgences Pédiatriques (GFRUP). Mortality and Neurologic Sequelae in Influenza-Associated Encephalopathy: Retrospective Multicenter PICU Cohort in France. Pediatr Crit Care Med. 2021 Nov 1;22(11):e582-e587. doi: 10.1097/PCC.0000000000002750. PMID: 33950890.	No	4	-
Pubmed	Aksoy E, Öztoprak Ü, Çelik H, Özdemir FMA, Özkan M, Kayılıoğlu H, Danış A, Kucur Ö, Kesici S, Uysal Yazıcı M, Azapağası E, Taşçı Yıldız Y, Ceylan N, Şenel S, Yüksel D. Acute necrotizing encephalopathy of childhood: a single-center experience. Turk J Med Sci. 2021 Apr 30;51(2):706-715. doi: 10.3906/sag-2102-47. PMID: 33754655; PMCID: PMC8203168.	Yes	-	-
Pubmed	Lim HY, Ho VP, Lim TC, Thomas T, Chan DW. Serial outcomes in acute necrotising encephalopathy of childhood: A medium and long term study. Brain Dev. 2016 Nov;38(10):928-936. doi: 10.1016/j.braindev.2016.05.002. Epub 2016 May 30. PMID: 27256511.	Yes	-	-
Pubmed	Bashiri FA, Al Johani S, Hamad MH, Kentab AY, Alwadei AH, Hundallah K, Hasan HH, Alshuaibi W, Jad L, Alrifai MT, Hudairi A, Al Sheikh R, Alenizi A, Alharthi NA, Abdelmagid TA, Ba-Armah D, Salih MA, Tabarki B. Acute Necrotizing Encephalopathy of Childhood: A Multicenter Experience in Saudi Arabia. Front Pediatr. 2020 Oct 9;8:526. doi: 10.3389/fped.2020.00526. PMID: 33163461; PMCID: PMC7581867.	No	5	-
Pubmed	Li K, Zhang T, Liu G, Jin P, Wang Y, Wang L, Xu M, Liu C, Liu Y, Zhou T, Xu Y, Yang Y, Fang B, Yang X, Liu C, Qian S. Plasma exchange therapy for acute necrotizing encephalopathy of childhood. Pediatr Investig. 2021 Jun 18;5(2):99-105. doi: 10.1002/ped4.12280. PMID: 34179705; PMCID: PMC8212728.	Yes	-	-
Pubmed	Muñoz-Osores E, Sánchez Ortiz N, Andresen Vásquez M, Ávila-Smirnow D, Valle Muñoz P, Barriga Gonzaga J. Encefalopatía necrotizante aguda asociada a influenza A [Influenza A-associated acute necrotizing encephalopathy]. Rev Chil Pediatr. 2020 Dec;91(6):941-946. Spanish. doi: 10.32641/rchped.vi91i6.1665. Epub 2020 Sep 24. PMID: 33861832.	No	2	-
Pubmed	Chang HP, Hsia SH, Lin JJ, Chan OW, Chiu CC, Lee EP. Early High-Dose Methylprednisolone Therapy Is Associated with Better Outcomes in Children with Acute Necrotizing Encephalopathy. Children (Basel). 2022 Jan 20;9(2):136. doi: 10.3390/children9020136. PMID: 35204857; PMCID: PMC8870393.	Yes	-	-
Pubmed	Okumura A, Mizuguchi M, Kidokoro H, Tanaka M, Abe S, Hosoya M, Aiba H, Maegaki Y, Yamamoto H, Tanabe T, Noda E, Imataka G, Kurahashi H. Outcome of acute necrotizing encephalopathy in relation to treatment with corticosteroids and	Yes	-	-

	gammaglobulin. Brain Dev. 2009 Mar;31(3):221-7. doi: 10.1016/j.braindev.2008.03.005. Epub 2008 May 5. PMID: 18456443.			
Pubmed	Zhu HM, Zhang SM, Yao C, Luo MQ, Ma HJ, Lei T, Yuan CH, Wu GF, Hu JS, Cai CQ, Liu ZS. The Clinical and Imaging Characteristics Associated With Neurological Sequelae of Pediatric Patients With Acute Necrotizing Encephalopathy. Front Pediatr. 2021 May 11;9:655074. doi: 10.3389/fped.2021.655074. PMID: 34046375; PMCID: PMC8144495.	Yes	-	-
Pubmed	Seo HE, Hwang SK, Choe BH, Cho MH, Park SP, Kwon S. Clinical spectrum and prognostic factors of acute necrotizing encephalopathy in children. J Korean Med Sci. 2010 Mar;25(3):449-53. doi: 10.3346/jkms.2010.25.3.449. Epub 2010 Feb 17. PMID: 20191046; PMCID: PMC2826728.	No	4	-
Pubmed	Garg RK, Paliwal VK, Gupta A. Encephalopathy in patients with COVID-19: A review. J Med Virol. 2021 Jan;93(1):206-222. doi: 10.1002/jmv.26207. Epub 2020 Jul 11. PMID: 32558956.	No	3	-
Pubmed	Bensaidane MR, Picher-Martel V, Émond F, De Serres G, Dupré N, Beauchemin P. Case Report: Acute Necrotizing Encephalopathy Following COVID-19 Vaccine. Front Neurol. 2022 Apr 29;13:872734. doi: 10.3389/fneur.2022.872734. PMID: 35572945; PMCID: PMC9099242.	No	2	-
Pubmed	Singh RR, Sedani S, Lim M, Wassmer E, Absoud M. RANBP2 mutation and acute necrotizing encephalopathy: 2 cases and a literature review of the expanding clinico-radiological phenotype. Eur J Paediatr Neurol. 2015 Mar;19(2):106-13. doi: 10.1016/j.ejpn.2014.11.010. Epub 2014 Dec 9. PMID: 25522933.	No	1	-
Pubmed	Ho JHY, Lee CYM, Chiong YK, Aoyama R, Fan LJ, Tan AHS, Han VX. SARS-CoV-2-Related Acute Necrotizing Encephalopathy of Childhood With Good Response to Tocilizumab in an Adolescent. Pediatr Neurol. 2023 Feb;139:65-69. doi: 10.1016/j.pediatrneurol.2022.11.010. Epub 2022 Nov 25. PMID: 36529001; PMCID: PMC9694347.	No	2	-
Pubmed	Okajima K, Hayakawa I, Tsuboi N, Shimura K, Ishiguro A, Abe Y. Early therapeutic plasma exchange may lead to complete neurological recovery in moderate to severe influenza-associated acute necrotizing encephalopathy. Brain Dev. 2022 Aug;44(7):492-497. doi: 10.1016/j.braindev.2022.03.004. Epub 2022 Mar 23. PMID: 35337691.	Yes	-	-
Pubmed	Koh JC, Murugasu A, Krishnappa J, Thomas T. Favorable Outcomes With Early Interleukin 6 Receptor Blockade in Severe Acute Necrotizing Encephalopathy of Childhood. Pediatr Neurol. 2019 Sep;98:80-84. doi: 10.1016/j.pediatrneurol.2019.04.009. Epub 2019 Apr 25. PMID: 31201070.	Yes	-	-
Pubmed	Kirkham FJ, Haywood P, Kashyape P, Borbone J, Lording A, Pryde K, Cox M, Keslake J, Smith M, Cuthbertson L, Murugan V, Mackie S, Thomas NH, Whitney A, Forrest KM, Parker A, Forsyth R, Kipps CM. Movement disorder emergencies in childhood. Eur J Paediatr Neurol. 2011 Sep;15(5):390-404. doi: 10.1016/j.ejpn.2011.04.005. Epub 2011 Aug 10. PMID: 21835657.	No	3	-
Pubmed	Lee VWM, Khoo TB, Teh CM, Heng HS, Li L, Yusof YLM, Yahaya NA, Dharshini S, Wong SW, Nickson T; ANE Malaysia Outcome Study Group. Factors associated with outcomes of severe acute necrotizing encephalopathy: A multicentre experience in Malaysia. Dev Med Child Neurol. 2023 Feb 7. doi: 10.1111/dmcn.15536. Epub ahead of print. PMID: 36748407.	Yes	-	-
Pubmed	Manara R, Franzoi M, Cogo P, Battistella PA. Acute necrotizing encephalopathy: combined therapy and favorable outcome in a new case. Childs Nerv Syst. 2006 Oct;22(10):1231-6. doi: 10.1007/s00381-006-0076-9. Epub 2006 Mar 14. PMID: 16816978.	No	2	-
Pubmed	Tabarki B, Thabet F, Al Shafi S, Al Adwani N, Chehab M, Al Shahwan S. Acute necrotizing encephalopathy associated with enterovirus infection. Brain Dev. 2013 May;35(5):454-7. doi: 10.1016/j.braindev.2012.07.001. Epub 2012 Jul 23. PMID: 22832063.	No	2	-
Pubmed	Thomas M, Swarnam K, Remadevi GS, Pillai AM. Acute Encephalitis Syndrome with an Unusual Diagnosis. J Trop Pediatr. 2020 Apr 1;66(2):228-230. doi: 10.1093/tropej/fmz058. PMID: 31504992.	No	2	-
Pubmed	Lee YJ, Hwang SK, Lee SM, Kwon S. Familial acute necrotizing encephalopathy with RANBP2 mutation: The first report in Northeast Asia. Brain Dev. 2017 Aug;39(7):625-628. doi: 10.1016/j.braindev.2017.02.005. Epub 2017 Mar 21. PMID: 28336122; PMCID: PMC7127085.	No	3	-

Pubmed	Dixon L, Varley J, Gontsarova A, Mallon D, Tona F, Muir D, Luqmani A, Jenkins IH, Nicholas R, Jones B, Everitt A. COVID-19-related acute necrotizing encephalopathy with brain stem involvement in a patient with aplastic anemia. Neurol Neuroimmunol Neuroinflamm. 2020 May 26;7(5):e789. doi: 10.1212/NXI.0000000000000789. PMID: 32457227; PMCID: PMC7286661.	No	2	-
Pubmed	Park YJ, Hwang JY, Kim YW, Lee YJ, Ko A. Radiological manifestation of familial acute necrotizing encephalopathy with RANBP2 mutation in a Far-East Asian family: Case report. Medicine (Baltimore). 2021 Mar 26;100(12):e25171. doi: 10.1097/MD.0000000000025171. PMID: 33761695; PMCID: PMC9282079.	No	2	-
Pubmed	Iizuka K, Suzuki K, Shiina T, Nakamura T, Funakoshi K, Hirata K. [Two adult patients with acute necrotizing encephalopathy following influenza virus infection]. Rinsho Shinkeigaku. 2020 Feb 27;60(2):157-161. Japanese. doi: 10.5692/clinicalneurol.cn-001381. Epub 2020 Jan 19. PMID: 31956199.	No	2	-
Pubmed	Bergamino L, Capra V, Biancheri R, Rossi A, Tacchella A, Ambrosini L, Mizuguchi M, Saitoh M, Marazzi MG. Immunomodulatory therapy in recurrent acute necrotizing encephalopathy ANE1: is it useful? Brain Dev. 2012 May;34(5):384-91. doi: 10.1016/j.braindev.2011.08.001. Epub 2011 Sep 25. PMID: 21945312.	No	2	-
Pubmed	Yoganathan S, Sudhakar SV, James EJ, Thomas MM. Acute necrotising encephalopathy in a child with H1N1 influenza infection: a clinicoradiological diagnosis and follow-up. BMJ Case Rep. 2016 Jan 11;2016:bcr2015213429. doi: 10.1136/bcr-2015-213429. PMID: 26759402; PMCID: PMC4716387.	No	2	-
Pubmed	Dale RC, Singh H, Troedson C, Pillai S, Gaikiwari S, Kozlowska K. A prospective study of acute movement disorders in children. Dev Med Child Neurol. 2010 Aug;52(8):739-48. doi: 10.1111/j.1469-8749.2009.03598.x. Epub 2010 Feb 12. PMID: 20163436.	No	3	-
Pubmed	Alawadhi A, Saint-Martin C, Bhanji F, Srour M, Atkinson J, Sébire G. Acute Hemorrhagic Encephalitis Responding to Combined Decompressive Craniectomy, Intravenous Immunoglobulin, and Corticosteroid Therapies: Association with Novel RANBP2 Variant. Front Neurol. 2018 Mar 12;9:130. doi: 10.3389/fneur.2018.00130. PMID: 29593631; PMCID: PMC5857578.	No	2	-
Pubmed	Fernández-Blázquez A, Castañón-Apilánez M, Álvarez-Argüelles ME, Sabater-Cabrera C, Rojo-Alba S, Boga JA, Morís de la Tassa G, Quindós Fernández B, Melón S. Neuroinvasion of influenza A/H3N2: a fatal case in an immunocompetent adult. J Neurovirol. 2019 Apr;25(2):275-279. doi: 10.1007/s13365-018-0690-9. Epub 2018 Nov 5. PMID: 30397824.	No	2	-
Pubmed	Işikay S. Influenza A (H1N1) Infection Associated Acute Necrotizing Encephalopathy in a Child With Periodic Lateralized Epileptiform Discharges. Pediatr Emerg Care. 2016 Dec;32(12):e14-e15. doi: 10.1097/PEC.0000000000000997. PMID: 27898637.	No	2	-
Pubmed	Neilson DE. The interplay of infection and genetics in acute necrotizing encephalopathy. Curr Opin Pediatr. 2010 Dec;22(6):751-7. doi: 10.1097/MOP.0b013e3283402bfe. PMID: 21610332.	No	1	-
Pubmed	Borah P, Deb PK, Chandrasekaran B, Goyal M, Bansal M, Hussain S, Shinu P, Venugopala KN, Al-Shar'i NA, Deka S, Singh V. Neurological Consequences of SARS-CoV-2 Infection and Concurrence of Treatment-Induced Neuropsychiatric Adverse Events in COVID-19 Patients: Navigating the Uncharted. Front Mol Biosci. 2021 Feb 18;8:627723. doi: 10.3389/fmolb.2021.627723. PMID: 33681293; PMCID: PMC7930836.	No	3	-
Pubmed	Wang KY, Singer HS, Crain B, Gujar S, Lin DD. Hypoxic-ischemic encephalopathy mimicking acute necrotizing encephalopathy. Pediatr Neurol. 2015 Jan;52(1):110-4. doi: 10.1016/j.pediatrneurol.2014.09.009. Epub 2014 Sep 22. PMID: 25447929.	No	3	-
Ovid	Appavu B, Foldes S, Fox J, Shetty S, Oh A, Bassal F, Marku I, Mangum T, Boerwinkle V, Neilson D, Kruer M. Treatment Timing, EEG, Neuroimaging, and Outcomes After Acute Necrotizing Encephalopathy in Children. J Child Neurol. 2021 Jun;36(7):517-524. doi: 10.1177/0883073820984063. Epub 2021 Jan 4. PMID: 33393838.	Yes	-	Yes
Ovid	Cleuziou P, Renaldo F, Renolleau S, Javouhey E, Tissieres P, Léger PL, Bergounioux J, Desguerre I, Dauger S, Levy M; Groupe Francophone de Réanimation et Urgences Pédiatriques (GFRUP). Mortality and Neurologic Sequelae in Influenza-Associated Encephalopathy: Retrospective Multicenter PICU Cohort in France. Pediatr Crit Care Med. 2021 Nov 1;22(11):e582-e587. doi: 10.1097/PCC.0000000000002750. PMID: 33950890.	No	-	Yes

Ovid	Park YJ, Hwang JY, Kim YW, Lee YJ, Ko A. Radiological manifestation of familial acute necrotizing encephalopathy with RANBP2 mutation in a Far-East Asian family: Case report. Medicine (Baltimore). 2021 Mar 26;100(12):e25171. doi: 10.1097/MD.0000000000025171. PMID: 33761695; PMCID: PMC9282079.	No	-	Yes
Ovid	Thomas M, Swarnam K, Remadevi GS, Pillai AM. Acute Encephalitis Syndrome with an Unusual Diagnosis. J Trop Pediatr. 2020 Apr 1;66(2):228-230. doi: 10.1093/tropej/fmz058. PMID: 31504992.	No	-	Yes
Ovid	Ho JHY, Lee CYM, Chiong YK, Aoyama R, Fan LJ, Tan AHS, Han VX. SARS-CoV-2-Related Acute Necrotizing Encephalopathy of Childhood With Good Response to Tocilizumab in an Adolescent. Pediatr Neurol. 2023 Feb;139:65-69. doi: 10.1016/j.pediatrneurol.2022.11.010. Epub 2022 Nov 25. PMID: 36529001; PMCID: PMC9694347.	No	-	Yes
Ovid	Okajima K, Hayakawa I, Tsuboi N, Shimura K, Ishiguro A, Abe Y. Early therapeutic plasma exchange may lead to complete neurological recovery in moderate to severe influenza-associated acute necrotizing encephalopathy. Brain Dev. 2022 Aug;44(7):492-497. doi: 10.1016/j.braindev.2022.03.004. Epub 2022 Mar 23. PMID: 35337691.	No	-	Yes
Ovid	Koh JC, Murugasu A, Krishnappa J, Thomas T. Favorable Outcomes With Early Interleukin 6 Receptor Blockade in Severe Acute Necrotizing Encephalopathy of Childhood. Pediatr Neurol. 2019 Sep;98:80-84. doi: 10.1016/j.pediatrneurol.2019.04.009. Epub 2019 Apr 25. PMID: 31201070.	Yes	-	Yes
Ovid	Lim HY, Ho VP, Lim TC, Thomas T, Chan DW. Serial outcomes in acute necrotising encephalopathy of childhood: A medium and long term study. Brain Dev. 2016 Nov;38(10):928-936. doi: 10.1016/j.braindev.2016.05.002. Epub 2016 May 30. PMID: 27256511.	Yes	-	Yes
CINAHL	Chang HP, Hsia SH, Lin JJ, Chan OW, Chiu CC, Lee EP. Early High-Dose Methylprednisolone Therapy Is Associated with Better Outcomes in Children with Acute Necrotizing Encephalopathy. Children (Basel). 2022 Jan 20;9(2):136. doi: 10.3390/children9020136. PMID: 35204857; PMCID: PMC8870393.	Yes	-	Yes
CINAHL	Cleuziou P, Renaldo F, Renolleau S, Javouhey E, Tissieres P, Léger PL, Bergounioux J, Desguerre I, Dauger S, Levy M; Groupe Francophone de Réanimation et Urgences Pédiatriques (GFRUP). Mortality and Neurologic Sequelae in Influenza-Associated Encephalopathy: Retrospective Multicenter PICU Cohort in France. Pediatr Crit Care Med. 2021 Nov 1;22(11):e582-e587. doi: 10.1097/PCC.0000000000002750. PMID: 33950890.	No	-	Yes
CINAHL	Thomas M, Swarnam K, Remadevi GS, Pillai AM. Acute Encephalitis Syndrome with an Unusual Diagnosis. J Trop Pediatr. 2020 Apr 1;66(2):228-230. doi: 10.1093/tropej/fmz058. PMID: 31504992.	No	-	Yes
CINAHL	Neilson DE. The interplay of infection and genetics in acute necrotizing encephalopathy. Curr Opin Pediatr. 2010 Dec;22(6):751-7. doi: 10.1097/MOP.0b013e3283402bfe. PMID: 21610332.	No	-	Yes
CINAHL	Koh JC, Murugasu A, Krishnappa J, Thomas T. Favorable Outcomes With Early Interleukin 6 Receptor Blockade in Severe Acute Necrotizing Encephalopathy of Childhood. Pediatr Neurol. 2019 Sep;98:80-84. doi: 10.1016/j.pediatrneurol.2019.04.009. Epub 2019 Apr 25. PMID: 31201070.	Yes	-	Yes
Embase	Cleuziou P, Renaldo F, Renolleau S, Javouhey E, Tissieres P, Léger PL, Bergounioux J, Desguerre I, Dauger S, Levy M; Groupe Francophone de Réanimation et Urgences Pédiatriques (GFRUP). Mortality and Neurologic Sequelae in Influenza-Associated Encephalopathy: Retrospective Multicenter PICU Cohort in France. Pediatr Crit Care Med. 2021 Nov 1;22(11):e582-e587. doi: 10.1097/PCC.0000000000002750. PMID: 33950890.	No	-	Yes
Embase	Zhu HM, Zhang SM, Yao C, Luo MQ, Ma HJ, Lei T, Yuan CH, Wu GF, Hu JS, Cai CQ, Liu ZS. The Clinical and Imaging Characteristics Associated With Neurological Sequelae of Pediatric Patients With Acute Necrotizing Encephalopathy. Front Pediatr. 2021 May 11;9:655074. doi: 10.3389/fped.2021.655074. PMID: 34046375; PMCID: PMC8144495.	Yes	-	Yes
Embase	Khan Q, Zhou L, Lightner D, Munnikhuysen S. Familial influenza associated acute necrotizing encephalopathy. Annals of Neurology. 2020. 88:SUPPL 25 (S144)	No	2	No
Embase	Thomas M, Swarnam K, Remadevi GS, Pillai AM. Acute Encephalitis Syndrome with an Unusual Diagnosis. J Trop Pediatr. 2020 Apr 1;66(2):228-230. doi: 10.1093/tropej/fmz058. PMID: 31504992.	No	-	Yes

Emba se	Lim HY, Ho VP, Lim TC, Thomas T, Chan DW. Serial outcomes in acute necrotising encephalopathy of childhood: A medium and long term study. Brain Dev. 2016 Nov;38(10):928-936. doi: 10.1016/j.braindev.2016.05.002. Epub 2016 May 30. PMID: 27256511.	Yes	-	Yes
Emba se	Ho JHY, Lee CYM, Chiong YK, Aoyama R, Fan LJ, Tan AHS, Han VX. SARS-CoV-2-Related Acute Necrotizing Encephalopathy of Childhood With Good Response to Tocilizumab in an Adolescent. Pediatr Neurol. 2023 Feb;139:65-69. doi: 10.1016/j.pediatrneurol.2022.11.010. Epub 2022 Nov 25. PMID: 36529001; PMCID: PMC9694347.	No	-	Yes
Emba se	Olubiyi OI, Zamora C, Jewells V, Hunter SE. Recurrent acute hemorrhagic necrotizing encephalopathy associated with RAN-binding protein-2 gene mutation in a pediatric patient. BJR Case Rep. 2022 Nov 1;8(6):20220019. doi: 10.1259/bjrcr.20220019. PMID: 36632547; PMCID: PMC9809908.	No	2	No
Emba se	Reppucci, D., & Datta, A. N. (2022). FIRES—Pathophysiology, therapeutical approach, and outcome. Zeitschrift für Epileptologie, 1-10.	No	3	No
Emba se	Okajima K, Hayakawa I, Tsuboi N, Shimura K, Ishiguro A, Abe Y. Early therapeutic plasma exchange may lead to complete neurological recovery in moderate to severe influenza-associated acute necrotizing encephalopathy. Brain Dev. 2022 Aug;44(7):492-497. doi: 10.1016/j.braindev.2022.03.004. Epub 2022 Mar 23. PMID: 35337691.	No	-	Yes
Emba se	Bensaidane MR, Picher-Martel V, Émond F, De Serres G, Dupré N, Beauchemin P. Case Report: Acute Necrotizing Encephalopathy Following COVID-19 Vaccine. Front Neurol. 2022 Apr 29;13:872734. doi: 10.3389/fneur.2022.872734. PMID: 35572945; PMCID: PMC9099242.	No	-	Yes
Emba se	Sebastian Werner, MD; Senyene Hunter, MD, PhD; Erin M. Finn, MD; William A. Rearick, MD, MPH; Irena Dujmovic Basuroski, MD, PhD; Qian-Zhou (JoJo) Yang, MD; Hannah Y. Coletti, MD, MPH. From ran(bp2)dom to Recurrent: A Case of Familial Acute Necrotizing Encephalopathy. Pediatrics (2022). 149 (1 Meeting Abstracts February 2022): 819.	No	2	No
Emba se	Shukla P, Mandalla A, Elrick MJ, Venkatesan A. Clinical Manifestations and Pathogenesis of Acute Necrotizing Encephalopathy: The Interface Between Systemic Infection and Neurologic Injury. Front Neurol. 2022 Jan 4;12:628811. doi: 10.3389/fneur.2021.628811. PMID: 35058867; PMCID: PMC8764155.	No	4	No
Emba se	Kushagra Singh, Sham Lohiy , Shubhangi Ganvir, Sachin Damke. Case of Acute Necrotizing Encephalitis Following Coronavirus Disease 2019 Infection-A Rare Presentation. Journal of Pediatric Neurology. DOI: 10.1055/s-0042-1756718.	No	2	No
Emba se	Hiraldo JDG, Domínguez-Mayoral A, García-Gómez FJ, Fouz-Rosón N, Rivas-Infante E, Cano MAM, Fernández AR, Morillo SG, Fernández NA, de León JAP, Mascarell GN, Lebrón CV. Central nervous system involvement in adult-onset relapsing hemophagocytic lymphohistiocytosis responsive to maintenance treatment with anakinra. J Neuroimmunol. 2021 Jun 15;355:577552. doi: 10.1016/j.jneuroim.2021.577552. Epub 2021 Apr 2. PMID: 33845282.	No	3	No
Emba se	Appavu B, Foldes S, Fox J, Shetty S, Oh A, Bassal F, Marku I, Mangum T, Boerwinkle V, Neilson D, Kruer M. Treatment Timing, EEG, Neuroimaging, and Outcomes After Acute Necrotizing Encephalopathy in Children. J Child Neurol. 2021 Jun;36(7):517-524. doi: 10.1177/0883073820984063. Epub 2021 Jan 4. PMID: 33393838.	Yes	-	Yes
Emba se	Park YJ, Hwang JY, Kim YW, Lee YJ, Ko A. Radiological manifestation of familial acute necrotizing encephalopathy with RANBP2 mutation in a Far-East Asian family: Case report. Medicine (Baltimore). 2021 Mar 26;100(12):e25171. doi: 10.1097/MD.0000000000025171. PMID: 33761695; PMCID: PMC9282079.	No	-	Yes
Emba se	Thepmankorn P, Bach J, Lasfar A, Zhao X, Souayah S, Chong ZZ, Souayah N. Cytokine storm induced by SARS-CoV-2 infection: The spectrum of its neurological manifestations. Cytokine. 2021 Feb;138:155404. doi: 10.1016/j.cyto.2020.155404. Epub 2020 Dec 11. PMID: 33360025; PMCID: PMC7832981.	No	3	No
Emba se	Bashiri FA, Al Johani S, Hamad MH, Kentab AY, Alwadei AH, Hundallah K, Hasan HH, Alshuaibi W, Jad L, Alrifai MT, Hudairi A, Al Sheikh R, Alenizi A, Alharthi NA, Abdelmagid TA, Ba-Armah D, Salih MA, Tabarki B. Acute Necrotizing Encephalopathy of Childhood: A Multicenter Experience in Saudi Arabia. Front Pediatr. 2020 Oct 9;8:526. doi: 10.3389/fped.2020.00526. PMID: 33163461; PMCID: PMC7581867.	No	-	Yes

Embase	Koh JC, Murugasu A, Krishnappa J, Thomas T. Favorable Outcomes With Early Interleukin 6 Receptor Blockade in Severe Acute Necrotizing Encephalopathy of Childhood. Pediatr Neurol. 2019 Sep;98:80-84. doi: 10.1016/j.pediatrneurol.2019.04.009. Epub 2019 Apr 25. PMID: 31201070.	No	-	Yes
Embase	Lim HY, Thomas T, Chan WS. Acute necrotising encephalopathy in childhood-epidemiology, radiological findings and outcomes. Archives of Disease in Childhood 2014 99 (A529) SUPPL. 2.	No	-	No
Embase	Dale RC, Singh H, Troedson C, Pillai S, Gaikiwari S, Kozlowska K. A prospective study of acute movement disorders in children. Dev Med Child Neurol. 2010 Aug;52(8):739-48. doi: 10.1111/j.1469-8749.2009.03598.x. Epub 2010 Feb 12. PMID: 20163436.	No	-	Yes
Scopus	Reppucci, D., & Datta, A. N. (2022). FIRES—Pathophysiology, therapeutical approach, and outcome. Zeitschrift für Epileptologie, 1-10.	-	-	Yes
Scopus	Zhu HM, Zhang SM, Yao C, Luo MQ, Ma HJ, Lei T, Yuan CH, Wu GF, Hu JS, Cai CQ, Liu ZS. The Clinical and Imaging Characteristics Associated With Neurological Sequelae of Pediatric Patients With Acute Necrotizing Encephalopathy. Front Pediatr. 2021 May 11;9:655074. doi: 10.3389/fped.2021.655074. PMID: 34046375; PMCID: PMC8144495.	-	-	Yes

Supplemental table 1: List of all identified citations, both included and excluded. Key to reasons not included: 1 – Screened out based on title or abstract because article was not a treatment/outcome focused case series or meta-analysis. 2 – Screened out based on title or abstract because article is a single case or case series with fewer than 3 patients. 3 – Screened out based on title or abstract because article was not about ANE. 4 – Screened in but was deemed ineligible because authors did not report individual patient data such that treatments could be individually linked to outcomes. 5 – Screened in but was deemed ineligible because authors did not report the timing of steroid treatment.
